# Prevalence and severity of low back- and pelvic girdle pain in pregnant Nepalese women

**DOI:** 10.1186/s12884-019-2398-0

**Published:** 2019-07-15

**Authors:** Ranjeeta Shijagurumayum Acharya, Anne Therese Tveter, Margreth Grotle, Malin Eberhard-Gran, Britt Stuge

**Affiliations:** 1 Department of Physiotherapy, Kathmandu University School of Medical Sciences, Kathmandu University Dhulikhel Hospital, Kavre, Nepal; 2 Institute of Clinical Medicine, University of Oslo, Oslo, Norway; 3 Division of Orthopaedic Surgery, Oslo University Hospital, Oslo, Norway; 4 Department of Physiotherapy, Faculty of Health Sciences, Oslo Metropolitan University, Oslo, Norway; 5 National Advisory Unit on Rehabilitation in Rheumatology, Department of Rheumatology, Diakonhjemmet Hospital, Oslo, Norway; 6 FORMI, Oslo University Hospital, Oslo, Norway; 7 Health Services Research Unit, Akershus University Hospital, Lørenskog, Norway; 8 Department for Infant Mental Health, Regional Center for Child and Adolescent Mental Health, Oslo, Eastern and Southern Norway Norway

**Keywords:** Pelvic girdle pain, Low back pain, Pelvic pain, Women’s health, Nepal, Pregnancy, Prevalence, Severity, Function, Impact

## Abstract

**Background:**

Low back pain (LBP) and pelvic girdle pain (PGP) are commonly reported during pregnancy and are known to affect pregnant women’s well-being. Still, these conditions are often considered to be a normal part of pregnancy. This study assesses the prevalence and severity of LBP and/or PGP among pregnant Nepalese women, as well as exploring factors associated with LBP and PGP.

**Methods:**

A cross-sectional study with successive recruitment of pregnant women was conducted at two district hospitals in Nepal from May 2016 to May 2017. The data was collected using self-reported questionnaires. Univariate and multivariate logistic regression were used to assess the associations between independent variables and LBP and/or PGP.

**Results:**

A total of 1284 pregnant women were included in the study. The reported prevalence of pregnancy-related LBP and/or PGP was 34%. Pain intensity was high with a mean score (standard deviation) of 6 (2). The median (25th-75th percentiles) disability scores according to the total Pelvic Girdle Questionnaire and Oswestry Disability Index were 20 (10–32) and 30 (21–38), respectively. Even though only 52% of the women believed that the pain would disappear after delivery, concern about LBP and/or PGP was reported to be low (median 2 (0–4) (Numeric Rating Scale 0–10)). In the final model for women with LBP and/or PGP the adjusted odds ratios were for body mass index (20–24, 25–30, > 30) 0.7 (95% confidence interval (CI), 0.44–1.21), 1.1 (95% CI, 0.66–1.83), and 1.5 (95% CI, 0.78–2.94) respectively, for pelvic organ prolapse symptoms 6.6 (95% CI, 4.93–8.95) and for women with educated husbands (primary or secondary, higher secondary or above) 1.1 (95% CI, 0.53–2.16) and 1.7 (95% CI, 0.84–3.47), respectively.

**Conclusions:**

Pregnant Nepalese women commonly report LBP and/or PGP. The women experienced low disability despite severe pain intensity and poor beliefs in recovery after delivery.

## Introduction

Global progress in maternal mortality rate reduction has placed emphasis on maternal morbidity, addressing maternal well-being and mental health [1, 2]. The World Health Organization’s Maternal Morbidity Working Group defined maternal morbidity as “any health condition attributed to and/or aggravated by pregnancy and childbirth that has a negative impact on the woman’s wellbeing” [2].

As the most common pregnancy-related musculoskeletal problems that impact pregnant women’s well-being are low back pain (LBP) and pelvic girdle pain (PGP), these conditions have garnered increasing interest worldwide [3–6]. LBP is defined as pain between the twelfth rib and the gluteal fold, while PGP is defined as pain experienced between the sacroiliac joint and the gluteal fold, or in the symphysis pubis [7]. The prevalence of pregnancy-related LBP and PGP varies from 20 to 80%, with the majority of studies reporting approximately 50% for LBP and/or PGP, and 20% for PGP specifically [4, 6–10].

Though not life-threatening conditions, pregnancy-related LBP and PGP may affect daily activities such as walking, working, sleep, and mood, thereby reducing quality of life [4, 5]. Commonly reported risk factors for pregnancy-related LBP and PGP are strenuous workload, frequent or prolonged torso flexion, previous history of LBP or PGP, and previous trauma to the pelvis [7, 8, 11]. Body mass index (BMI), parity, and depression are also found to be associated with LBP and PGP [6, 9, 10].

Nepal’s main source of income is agriculture, with 76% of households involved in agricultural activities, and 84% of women involved in agricultural work [12, 13]. The increase in labor migration among Nepal’s men has led to a triple role for women, who are involved in reproduction, agricultural production, and household work [14, 15]. The number of Nepalese women faced with physically strenuous work, little time for rest, and reduced family support, even during pregnancy, is high [16].

Pregnancy-related LBP and PGP are not generally regarded as serious complications and can thus be overlooked by healthcare professionals [4]. Nevertheless, disability and negative psychological effects related to LBP and PGP have been reported in many countries [4–6, 9, 10, 17–19]. A lack of awareness of the impairment caused by these musculoskeletal complaints can result in poor management of pregnant women. A multinational study showed variation in the prevalence, severity, and concern surrounding pregnancy-related LBP and PGP across different countries [4]. Societal attitudes, ethnicity, and cultural beliefs might influence how pregnancy-related LBP and/or PGP are perceived [20, 21]. Most studies on pregnancy-related LBP and PGP have been conducted in developed countries, and it seems that these conditions are often overlooked in developing countries [22].

To our knowledge, the prevalence and severity of pregnancy-related LBP and PGP have never been examined in Nepal [23]. Thus, the primary aim of this study was to assess the prevalence and severity of LBP and PGP in pregnant Nepalese women. A secondary aim was to determine the factors that influence LBP and PGP during pregnancy.

## Methods

### Study design and setting

A cross-sectional study with successive recruitment of pregnant women was conducted at two district hospitals in Nepal from May 2016 to May 2017. KIST Teaching Hospital in Lalitpur district is situated in the capital city Kathmandu, whereas Kathmandu University Dhulikhel Hospital (KUDH), in Kavreplanchowk district, is located 30 km northeast from Kathmandu. KIST and KUDH hospitals see about 1200 and 3500 live births each year, respectively.

### Participants

Pregnant women attending antenatal control at KUDH and KIST hospitals were eligible for inclusion in the study if they were willing to participate, had no history of spinal fracture or surgery, and could understand and speak Nepali. Oral and written consent was obtained from women who agreed to participate in the study. One of two reasearch assistants collected and recorded data on a Samsung tablet with open data kit software.

Ethical approval was obtained from the Norwegian Regional Ethics Committee (REK Nord, 2015/2209), the Nepal Health Research Council Ethical Review Board (112/2016), and the Institutional Review Committee of Kathmandu University School of Medical Sciences/Dhulikhel Hospital (25/16).

### Measurements

Participants completed questionnaires on sociodemographic, pregnancy, and workload characteristics. Sociodemographic information included: age (years), height (cm), weight (kg), ethnicity, women’s and husbands education in years (no education, primary ≤ 5, lower secondary 6–8, secondary 9–10, higher secondary 11–12, bachelor and above ≥ 13), monthly income in United States dollars (USD,$) (no income, less than $76, $76–153, > $153), occupation, marital status (living with husband, husband working away from home, divorced), type of family (nuclear: husband and children; joint: parents-in-law, husband, and children; extended: grandparents, parents-in-law, husband, and children), number of family members, and household work (washing/cleaning/cooking, child care, animal care, fetching water). Pregnancy characteristics included number of pregnancies, parity, and gestation (weeks). Workload information included waking hours, hours of rest during work, types of field work (farming, grass cutting, branch cutting, fetching water), working positions (sitting or squatting, standing or bending forward), and hours of prolonged sitting or standing.

The registration of LBP and PGP was done using a body chart. Participants who reported musculoskeletal pain were asked to indicate the location of their pain on a body chart, which was then validated by having the women point to the site of the pain on their body. If the women pointed to their lower back and/or pelvis, they were considered to have LBP and/or PGP. A physiotherapist then performed further clinical examination on women reporting LBP and/or PGP [7, 24, 25].

The women who reported LBP and/or PGP also responded to questions regarding pain intensity (numeric rating scale, 0 (no pain) to 10 (the most severe pain)) [26, 27], pain frequency (on some days, most days, every day), specific pelvic pain locations (sacrum, sacroiliac joints, and symphysis pubis), whether the LBP and/or PGP limited their usual activities or changed their daily routine for more than 1 day (no/yes) [28], whether they were concerned about the LBP and/or PGP (numeric rating scale, 0 (not concerned) to 10 (extremely concerned), and whether they believed that the LBP and/or PGP would disappear after delivery (no/yes).

Reliable and valid Nepalese versions of outcome measures were used to assess the severity of activity limitations and symptoms of pregnancy-related LBP and PGP. The Oswestry Disability Index (ODI), version 2 [29, 30], consists of 10 items with scores from 0 to 5, describing an increasing degree of difficulty. The Pelvic Girdle Questionnaire (PGQ) comprises 25 items with scores on a 4-point descriptive scale from 0 to 3, describing an increasing degree of symptoms and activity limitations [31–33]. The total sum score for both the ODI and the PGQ is expressed as a percentage from 0 to 100 (severe disability).

The women completed questionnaires on pelvic organ prolapse (POP) symptoms, urinary incontinence (UI), and depression symptoms. The POP symptom score (POP-SS) ranges from 0 to 28, with higher scores indicating more severe symptoms [34]. The International Consultation on Incontinence Questionnaire-UI Short Form (ICIQ-UI SF) score ranges from 0 to 21, with higher scores representing more severe UI symptoms [35]. The short five-item version of the Edinburgh Depression Scale (EDS-5) has four alternative answers to each question, scored from 0 to 3, adding up to a maximum score of 15 to represent the highest severity of depression [36].

### Statistical analysis

Continuous variables with normal distribution are presented as mean (standard deviation (SD)) or median (interquartile range (iqr), 25th and 75th percentiles) if skewed. Categorical data are presented as numbers and percentages.

To assess the associations between independent variables and LBP and/or PGP, univariate and multivariate logistic regression with odds ratios (OR) and 95% confidence intervals (CIs) were used. The authors planned to include 20 independent variables to assess these associations, requiring at least 200 participants with pregnancy-related LBP and/or PGP. Prior to logistic regression analysis, the independent variables were tested for collinearity, and correlations greater than 0.7 were considered to be one-dimensional. Following this rule, potential univariate associations for 20 independent variables with the outcome of LBP and/or PGP were analyzed using Mann-Whitney U or Chi-square tests, depending on whether variables were continuous or categorical. Variables with *p*-values less than or equal to 0.25 were included in the multivariate regression model.

In order to enhance interpretability, the variables are presented in the following categories: age: ≤ 21, 22–24, 25–27, ≥ 28 years; weeks of gestation: 1–12, 13–28, 29–40; BMI: < 20, 20–24, 25–30, > 30; education: no education, primary-secondary (5–10 years), higher secondary and above (≥ 11 years); monthly income: no income, ≤ $153, and > $153; child care (no/yes), fetching water (no/yes), field work (no/yes), rest during work (no/yes); POP-SS: no symptoms vs ≥ 3 symptoms of POP (75th percentile); ICIQ-UI SF: no UI or reported UI; and EDS-5: < 5 or ≥ 5 symptoms of depression. A backward strategy was used in the multivariate modelling. Variables with *p*-values greater than or equal to 0.05 were excluded. Variables in the final model were checked for pair-wise interactions. The Statistical Package for the Social Sciences (SPSS) version 22 was used for statistical analysis.

## Results

A total of 1284 pregnant women (54% from KUDH and 46% from KIST) were included in the study (Table 1). The mean age of the participants was 25 (SD 4) years, with a mean of 24 (SD 10) gestation weeks. Fifty-eight percent of the women were nulliparous. More than half of the women had secondary education, but only 14% had a monthly income. Fifty-two percent of the participants had to take care of animals and fetch water in addition to doing usual household work. Only one**-**third of the women reported taking breaks to rest while working, and the reported mean hours of rest per day was 1 (SD 1). Fifteen percent (*n* = 195) of the participants were involved in field work. Farming and grass cutting were the most common types of field work, and most of the women’s working hours were spent either sitting, standing, or bending forward, with a mean of 3 (SD 2) hours of prolonged standing.

**Table 1:** Characteristics of pregnant women included in the study (*n* = 1284)

Variables	Value
Age, years, mean (SD)	25 (4)
Gestational week, mean (SD)^a^	24 (10)
Number of pregnancies, median (iqr)	2 (1–2)
Parity, n (%)	
0	751 (58)
1	461 (36)
≥ 2	72 (6)
Body mass index, kg/m^2^, mean (SD)^b^	25 (4)
Marital status, n (%)	
Living with husband	1161 (90)
Husband works away from home	121 (10)
Divorce	2 (0)
Husband residing abroad, n (%)	85 (7)
Type of family, n (%)	
Nuclear (husband and children)	593 (46)
Joint (parents-in-law, husband, and children)	645 (50)
Extended (grandparents, parents-in-law, husband, and children)	46 (4)
Number of family members, mean (SD)	5 (3)
Ethnicity, n (%)	
Brahmin	244 (19)
Chhetri	231 (18)
Newar	283 (22)
Tamang	267 (21)
Magar	54 (4)
Dalit	74 (6)
Others	131 (10)
Participant’s education, years, n (%)	
No education	113 (9)
Primary (≤ 5)	148 (11)
Lower secondary (6–8)	174 (13)
Secondary (9–10)	303 (24)
Higher secondary (11–12)	341 (27)
Bachelor and above (≥ 13)	205 (16)
Husbands education, years, n (%)	
No education	60 (5)
Primary (≤ 5)	131 (10)
Lower secondary (6–8)	195 (15)
Secondary (9–10)	330 (26)
Higher secondary (11–12)	338 (26)
Bachelor and above (≥ 13)	230 (18)
Women with monthly income, USD, n (%)	
No income	1100 (86)
< 76	55 (4)
76–153	72 (6)
> 153	57 (4)
Husband with monthly income, USD, n (%)	
No income	862 (67)
< 76	24 (2)
76–153	150 (12)
> 153	248 (19)
Occupation, n (%)	
Housewife	1160 (90)
Agriculture	54 (4)
Business (owns a shop, cattle rearing etc.)	114 (9)
Employed (government and private)	124 (10)
Others (labor, student)	26 (2)
Household work, n (%)	
Washing, cleaning, cooking	1284 (100)
Child care	451 (35)
Animal care	207 (16)
Fetching water	463 (36)
Field work, n (%)	195 (15)
Waking hours in a day, mean (SD)	15 (2)
Rest taken during work, n (%)	436 (34)
LBP and/or PGP in past 4 weeks, n (%)	432 (34)
POP-SS, median (iqr)	0 (0–3)
ICIQ-UI SF, median (iqr)	0 (0–0)
EDS-5, median (iqr)	2 (0–4)

*SD* Standard deviation; Interquartile range (iqr; percentiles 25th – 75th), *USD* United States dollar, *LBP* Low back pain, *PGP* Pelvic girdle pain, *POP-SS* Pelvic organ prolapse symptom score, *ICIQ-UI SF* International consultation on incontinence questionnaire- urinary incontinence short form, *EDS-5* Edinburgh depression scale 5-item version

^a^missing number: 15

^b^missing number: 7

The reported prevalence of pregnancy-related LBP and/or PGP was 34% (Table 1). The sacrum and sacroiliac joints were the most commonly reported sites of pelvic pain, and only 7% of the women had pain in all three of the pelvic joints (Table 2). The clinical examination was limited, as many of the women refused to perform all clinical tests. The posterior pelvic pain provocation test was, however, performed in 379 (88%) women, and 73% had a positive result. Among the participants reporting LBP and/or PGP, evening pain intensity was reported to be high, with a mean of 6 (SD 2), while the total PGQ and ODI scores showed low and moderate disability, respectively. Twenty-four percent of the women had pain most days, and one-third reported a limitation of their ability to perform their usual activities for more than 1 day due to LBP and/or PGP. Concern about LBP and/or PGP was reported to be low (median 2 (iqr 0–4)). Almost 50% of the participating women were either unsure or did not believe that their pain would disappear after delivery.

**Table 2:** Descriptives of self-reported low back pain (LBP) and/or pelvic girdle pain (PGP) (*n* = 432)

Variables	Value
LBP/PGP frequency, n (%)	
Some days	285 (66)
Most days	102 (24)
Every day	45 (10)
Limit daily activities, n (%)	139 (32)
Pelvic pain location, n (%)	
Symphysis pubis	55 (13)
Right sacroiliac joint	262 (61)
Left sacroiliac joint	250 (58)
Sacrum	347 (80)
Pain intensity (NRS 0–10) in past 4 weeks, mean (SD)	5 (2)
Evening pain intensity (NRS 0–10) in past 4 weeks, mean (SD)	6 (2)
Concern about LBP and/or PGP (NRS 0–10), median (iqr)	2 (0–4)
Pain will disappear after delivery, n (%)	
Yes	224 (52)
No	117 (27)
Uncertain	89 (21)
Pelvic Girdle Questionnaire (PGQ), (0–100)	
PGQ total score, median (iqr)	20 (10–32)
PGQ activity subscale score, median (iqr)	21 (9–32)
PGQ symptom subscale score, median (iqr)	20 (7–33)
Oswestry Disability Index (ODI) (0–100), median (iqr)	30 (21–38)

*SD* Standard deviation; Interquartile range (iqr; percentiles 25th–75th), *NRS* Numeric rating scale

The univariate analyses showed a higher likelihood of pregnancy-related LBP and/or PGP in women with increased BMI, increasing weeks of gestation, higher education and higher monthly income, symptoms of POP, UI and depression, women who fetched water, and those whose husbands had a higher education and a higher monthly income (Table 3). The multivariate regression analysis showed significant associations in women with increased BMI, with husbands with higher education, and for women with POP symptoms (Table 4). No interactions were observed in the multivariate model. The strongest association was found between reported POP symptoms and LBP and/or PGP.

**Table 3:** Univariate analysis of factors associated with and without pregnancy-related low back pain (LBP) and/or pelvic girdle pain (PGP)

Variables	With LBP and/or PGP (*n* = 432)	Without LBP and/or PGP (*n* = 852)	Odds ratio (95% confidence interval)	*P*-value
Age, years, n (%)				
≤ 21	92 (21)	238 (28)	–	0.08
22–24	133 (31)	243 (28)	1.4 (1.03–2.00)	
25–27	99 (23)	177 (21)	1.5 (1.03–2.04)	
≥ 28	108 (25)	194 (23)	1.4 (1.03–2.02)	
Weeks of gestation, n (%)				
1–12	52 (12)	127 (15)	–	0.09
13–28	208 (49)	430 (51)	1.2 (0.82–1.70)	
29–40	168 (39)	284 (34)	1.4 (0.99–2.10)	
Body mass index, n (%)				
< 20	36 (10)	63 (9)	–	< 0.001
20–24	142 (38)	371 (50)	0.7 (0.43–1.05)	
25–30	159 (42)	259 (35)	1.1 (0.68–1.69)	
> 30	39 (10)	45 (6)	1.5 (0.84–2.74)	
Participant’s education, years, n (%)				
No education	25 (6)	88 (10)	–	0.003
Primary-secondary (5–10)	200 (46)	425 (50)	1.7 (1.03–2.66)	
Higher secondary and above (≥11)	207 (48)	339 (40)	2.2 (1.33–3.46)	
Husbands education, years, n (%)				
No education	15 (3)	45 (5)	–	0.004
Primary- secondary (5–10)	199 (46)	457 (54)	1.3 (0.71–2.40)	
Higher secondary and above (≥ 11)	218 (51)	350 (41)	1.9 (1.02–3.43)	
Participant’s monthly income, USD, n (%)				
No income	352 (81)	748 (88)	–	0.009
≤ 153	56 (13)	71 (8)	1.7 (1.16–2.43)	
> 153	24 (6)	33 (4)	1.6 (0.90–2.65)	
Husbands monthly income, USD, n (%)				
No income	265 (61)	597 (70)	–	0.007
≤ 153	68 (16)	106 (12)	1.4 (1.03–2.03)	
> 153	99 (23)	149 (18)	1.5 (1.11–2.01)	
Child care, n (%)				
No	291 (67)	542 (64)	–	0.18
Yes	141 (33)	310 (36)	0.8 (0.66–1.08)	
Fetching water, n (%)				
No	260 (60)	561 (66)	–	0.05
Yes	172 (40)	291 (34)	1.3 (1.00–1.62)	
Field work, n (%)				
No	356 (82)	733 (86)	–	0.09
Yes	76 (18)	119 (14)	1.3 (0.96–1.80)	
Rest during work, n (%)				
No	271 (63)	577 (68)	–	0.07
Yes	161 (37)	275 (32)	1.2 (0.98–1.59)	
POP-SS, n (%)				
0–2 (no symptoms of POP)	209 (48)	731 (86)	–	< 0.001
≥ 3 (symptoms of POP)	223 (52)	121 (14)	6.4 (4.92–8.34)	
ICIQ-UI SF, n (%)				
0- No UI	333 (77)	723 (85)	–	0.003
1- Reported UI	99 (23)	129 (15)	1.7 (1.24–2.23)	
EDS-5, n (%)				
< 5 - no symptoms of depression	309 (72)	714 (84)	–	< 0.001
≥ 5 - symptoms of depression	123 (28)	138 (16)	2.1 (1.56–2.72)	

*USD* United States dollar, *POP-SS* Pelvic organ prolapse symptom score, ≥ 3 (symptoms of POP); 75th percentile, *ICIQ-UI SF* International consultation on incontinence questionnaire-urinary incontinence short form, *EDS-5* Edinburgh depression scale 5-item version (cut off ≥ 5 for symptoms of depression)

**Table 4:** Multivariate analysis of factors associated with pregnancy-related low back pain (LBP) and/or pelvic girdle pain (PGP)

Variables	Odds ratio (95% confidence interval)	*P*-value
Age, years, n (%)		
≤ 21	–	0.54
22–24	1.3 (0.86–1.85)	
25–27	1.3 (0.84–1.90)	
≥ 28	1.3 (0.87–1.96)	
Body mass index, n (%)		
< 20	–	0.01
20–24	0.7 (0.44–1.21)	
25–30	1.1 (0.66–1.83)	
> 30	1.5 (0.78–2.94)	
Husbands education, years, n (%)		
No education	–	0.003
Primary- secondary (5–10)	1.1 (0.53–2.16)	
Higher secondary and above (≥ 11)	1.7 (0.84–3.47)	
POP-SS, n (%)		
0–2 (no symptoms of POP)	–	< 0.001
≥ 3 (symptoms of POP)	6.6 (4.93–8.95)	

Adjusted for age in the multivariate analysis

*POP-SS* Pelvic organ prolapse symptom score, ≥ 3 (symptoms of POP); 75th percentile

## Discussion

In this one-year prevalence study we found that LBP and/or PGP were commonly reported in pregnant Nepalese women. Though the reported pain intensity was high, disability and impact on daily life was found to be low. Only half of the participants believed that their pain would disappear after delivery. Multivariate analysis showed that women with increased BMI, POP symptoms, and husbands with higher education were significantly more likely to have pregnancy-related LBP and/or PGP.

The prevalence of LBP and/or PGP is consistent with a recent study from Western Nepal, in which back pain was reported as one of the most common complications during pregnancy [23]. A comparable study from another developing country also reported that LBP and PGP were commonly reported during pregnancy [17]. Still, the reported prevalence of pregnancy-related LBP and/or PGP in our study was found to be lower (34%) compared to studies in developed countries (45–86%) [4, 6, 8–10]. Possible explanations for the lower prevalence could be that most of our participants were pregnant for the first time, with a mean gestation of 24 weeks, compared to participants in other studies who were in their third trimester [4, 6, 10]. One or more previous deliveries and increasing numbers of weeks of gestation have been reported to increase the risk of LBP and/or PGP [9, 10, 17, 37].

Prevalence rates might also be influenced by the way data is collected. Data on LBP and PGP are commonly collected using questionnaires [4, 38]. Optimally, classification of LBP and PGP should be done through a thorough clinical examination [7]. Unfortunately, we were not able to clinically examine all the women. We based our classification on a validated body chart by asking the women to point to the pain on their body site. Body charts have previously been used to determine LBP and/or PGP; however, in contrast to our study, many studies lack a validation of the body chart [4, 10].

Compared to a recent international study, the women in our study showed higher pain intensity than women in the United States, Norway, and Sweden, but lower than women in the United Kingdom [4]. The women in the United Kingdom had lower education than the women studied in the other countries. The majority of the women in our sample also had lower education levels, which could be one reason for their reported high pain intensity, as education level has been found to be associated with severity of symptoms [39].

Despite high pain intensity, low disability scores were observed. Reported disability rates were much higher in pregnant women in Scandinavian countries, the United Kingdom, and the United States [4]. This result could be influenced by the differences in gestational period among the various groups of women studied, nevertheless, the inconsistency between pain intensity and disability is surprising. Only 10% of our sample reported LBP and/or PGP every day, and only one-third of the women reported that they had to limit their daily activities. These rates are lower than those seen in the study by Gutke et al. [4] Pain tolerance has been shown to be strongly associated with ethnic differences [20]. The differences in impact on daily life might be explained by the way Nepalese women adapt to pain or adopt different ways of coping with pain. Another possible explanation could be the neglect of health problems in Nepal due to the challenges of survival and meeting basic needs [16].

The participants were not very concerned about their LBP and/or PGP, even though only half of them believed that their pain would disappear after delivery. This belief in persistent pain postpartum could be due to lack of knowledge regarding the prognosis and recovery for LBP and PGP after delivery [40]. A study from Nepal reported the challenges faced by healthcare providers in providing information to patients with low education [41]. The lower educational level of our sample and lack of information regarding common pregnancy complaints might have led to their belief in persistent LBP and/or PGP postpartum.

We found that women with a higher BMI were more likely to report LBP and/or PGP. This finding is consistent with several previous studies [6, 9, 10, 42], although one study [43] reported no association. One surprising finding in the current study was that women with educated husbands were positively associated with pregnancy-related LBP and/or PGP. The majority of the women in our study were unemployed, and since highly educated husbands probably work away from home, it is likely that these women are responsible for household work, which might result in an increased strenuous physical workload influencing LBP and PGP [8, 11]. Our finding is supported by another study from Nepal where the overburden of physical work on women is reported to be due to the migration of their husbands, either to urban centers or abroad, for employment [15]. Half of the women in our study had to fetch water and take care of animals, in addition to household chores, and 15% of the studied women were also involved in field work. Heavy physical workload might be a reason for Nepalese women reporting a significantly higher prevalence of backache compared to Nepalese men [44].

POP symptoms were also found to be associated with pregnancy-related LBP and/or PGP in our study. LBP, PGP, and POP are reported to be influenced by weakness and laxity of the muscles and their supporting structures [45, 46]. During pregnancy the laxity of pelvic ligaments is exacerbated due to the release of the hormone relaxin [47]. Even though increased concentration of relaxin is seen during pregnancy, a positive association has not been established between pregnancy-related PGP and relaxin [48]. Dufour et al. [49] reported high correlations between pelvic organ dysfunction and lumbo-pelvic pain, whereas Stuge at al. [50] reported no evidence for impaired pelvic floor muscle function in postpartum women with PGP, though a tendency towards POP was reported. We used POP-SS, which is a self-reported outcome measure for identifying POP symptoms, and as one of the questions is related to LBP this might influence the association.

Our univariate model showed significant associations of LBP and/or PGP with women with higher education and income, women whose husbands had higher income, weeks of gestation, workload (fetching water, rest during work, field work), and UI and depression symptoms. However, in contrast to previous studies, none of these variables were associated in our multivariate model [9–11, 19, 39, 51]. Compared to most studies performed in developed countries, our study might be influenced by other social and ethnic factors [20, 21]. More studies in developing countries are needed to increase the knowledge related to these factors.

A strength of our study is the large sample size of 1284 pregnant women. Further, to gain a broader representation of Nepal’s population, data was collected from two hospitals in different districts that care for patients from rural (KUDH hospital) and urban (KIST hospital) areas. Still, the results from our study population might not be generalized to women living in remote areas. Another strength is that we used reliable and valid outcome measures for assessing LBP and PGP, allowing us to compare our results with those from other studies. To our knowledge, the prevalence, severity, and associations of pregnancy-related LBP and/or PGP have not previously been assessed in Nepal using standardized questionnaires. The use of open data kit software for data collection led to very little missing data, further strengthening our study. One possible limitation is that the questionnaires were administrated by a research assistant, however, since most of the participants had low levels of education, oral administration was needed to obtain data.

The present study shows that LBP and PGP are prevalent in pregnant Nepalese women and that women experience severe pain intensity alongside a belief in persistent pain post-partum. In developing countries like Nepal, LBP and PGP might be overlooked or considered to be normal complaints during pregnancy [22]. Nepalese women contribute significantly to agricultural and household work, even during pregnancy, and good physical health is needed [15, 16, 44]. Hence, our study highlights the need to address the complaints of LBP and PGP during pregnancy, and for health care providers to deliver timely information and treatment to prevent long-lasting pain.

## Conclusions

LBP and PGP are prevalent in pregnant Nepalese women. Despite severe pain intensity, the women experienced low disability. Women with increased BMI, POP symptoms, and husbands with higher education were significantly associated with LBP and/or PGP. Women’s belief in poor recovery of LBP and PGP after delivery indicates a need for assessment and information about musculoskeletal complaints in pregnant women in Nepal.

## Data Availability

The datasets used and/or analysed during the current study are available from the corresponding author on request.

## Abbreviations

BMI: Body mass index
CI: Confidence interval
EDS-5: Edinburgh Depression Scale-5
ICIQ-UI SF: International Consultation on Incontinence Questionnaire-Urinary Incontinence Short Form
KUDH: Kathmandu University Dhulikhel Hospital
LBP: Low back pain
NRS: Numeric Rating Scale
ODI: Oswestry Disability Index
PGP: Pelvic girdle pain
PGQ: Pelvic Girdle Questionnaire
POP: Pelvic organ prolapse
POP-SS: Pelvic Organ Prolapse Symptom Score
SD: Standard deviation
UI: Urinary incontinence
USD: United States dollar
